# Interleukin-4 Alters Early Phagosome Phenotype by Modulating Class I PI3K Dependent Lipid Remodeling and Protein Recruitment

**DOI:** 10.1371/journal.pone.0022328

**Published:** 2011-07-25

**Authors:** Sandra de Keijzer, Marjolein B. M. Meddens, Dilek Kilic, Ben Joosten, Inge Reinieren-Beeren, Diane S. Lidke, Alessandra Cambi

**Affiliations:** 1 Department of Tumor Immunology, Nijmegen Centre for Molecular Life Sciences, Radboud University Nijmegen Medical Centre, Nijmegen, The Netherlands; 2 Department of Pathology and Cancer Research and Treatment Center, University of New Mexico, Albuquerque, New Mexico, United States of America; Yale Medical School, United States of America

## Abstract

Phagocytosis is a complex process that involves membranelipid remodeling and the attraction and retention of key effector proteins. Phagosome phenotype depends on the type of receptor engaged and can be influenced by extracellular signals. Interleukin 4 (IL-4) is a cytokine that induces the alternative activation of macrophages (MΦs) upon prolonged exposure, triggering a different cell phenotype that has an altered phagocytic capacity. In contrast, the direct effects of IL-4 during phagocytosis remain unknown. Here, we investigate the impact of short-term IL-4 exposure (1 hour) during phagocytosis of IgG-opsonized yeast particles by MΦs. By time-lapse confocal microscopy of GFP-tagged lipid-sensing probes, we show that IL-4 increases the negative charge of the phagosomal membrane by prolonging the presence of the negatively charged second messenger PI(3,4,5)P3. Biochemical assays reveal an enhanced PI3K/Akt activity upon phagocytosis in the presence of IL-4. Blocking the specific class I PI3K after the onset of phagocytosis completely abrogates the IL-4-induced changes in lipid remodeling and concomitant membrane charge. Finally, we show that IL-4 direct signaling leads to a significantly prolonged retention profile of the signaling molecules Rac1 and Rab5 to the phagosomal membrane in a PI3K-dependent manner. This protracted early phagosome phenotype suggests an altered maturation, which is supported by the delayed phagosome acidification measured in the presence of IL-4. Our findings reveal that molecular differences in IL-4 levels, in the extracellular microenvironment, influence the coordination of lipid remodeling and protein recruitment, which determine phagosome phenotype and, eventually, fate. Endosomal and phagosomal membranes provide topological constraints to signaling molecules. Therefore, changes in the phagosome phenotype modulated by extracellular factors may represent an additional mechanism that regulates the outcome of phagocytosis and could have significant impact on the net biochemical output of a cell.

## Introduction

The constant threat posed by pathogens and cell debris is tackled by phagocytosis, the process through which cells engulf and destroy dangerous material [1]. Phagocytosis is a complex process that can be divided into the formation of phagosomes and their subsequent maturation that enables them for pathogen elimination and antigen presentation.

The cell environment can play an important role in regulating the outcome of the phagocytic process. A variety of self and pathogen derived signals can influence phagocytosis and the microbicidal activity of specialized phagocytes such as macrophages (MΦs) and neutrophils [2]–[4]. Among these signals is the type I cytokine Interleukin 4 (IL-4), mainly produced by T helper 2 cells and mast cells and involved in a variety of (patho)physiological events ranging from tissue adhesion and inflammation, to specificity of immunoglobulin class switching and the regulation of immune responses to allergens and parasites [5].

In the past decade, IL-4 has been widely studied for its capacity to induce the so called ‘alternative activation’ of MΦs thereby triggering a different phenotype than the IFN-γ mediated classical activation [6]. In particular, IL-4 activated MΦs are characterized by a potentiated production of lypopolysaccharide-stimulated cytokines and chemokines and enhanced endocytosis [7]. In contrast, the effects of IL-4 on phagocytosis in MΦs remain controversial as both enhanced and decreased phagocytic capacity were observed in IL-4-treated MΦs [8]–[11]. The existence of multiple phagocytosis modes, characterized by the engagement of specific receptor repertoires with subsequent diverse interplay [1], may explain the opposing effects reported for IL-4. Moreover, after days of IL-4 treatment it is difficult to ascribe changes in phagocytic capacity to direct IL-4 signaling or rather to signaling as a results of IL-4 induced gene expression. Consequently, the effect of phagocytic stimuli on intracellular signaling of IL-4-treated MΦs is poorly defined. Therefore, new insight into the effects of a direct IL-4 signaling on phagocytosis is needed.

One of the best characterized phagocytic processes is the Fcγ receptor-mediated phagocytosis in MΦs [12]. Here, the engulfment of IgG-opsonized particles is initiated by clustering of Fc-receptors on the surface of the phagocytic cell. Receptor stimulation induces activation of Src family kinases, which phosphorylates tyrosine residues at the immunoreceptor tyrosine-based activation motif (ITAM) that serves as docking site for the kinase Syk [13], [14]. Activated Syk initiates several downstream signaling pathways, including the class I phosphatidylinositol 3′-kinase (PI3K) pathway, which are still only partially understood (reviewed in [15]). Following formation, the phagosomes acquire microbicidal properties through a maturation process that involves a series of interactions with endocytic compartments, eventually fusing with lysosomes to form a phagolysosome [16].

Recently, it has become clear that phagocytosis involves extensive lipid remodeling [17], [18]. Lipids assemble into microdomains that can act as signaling platforms and confer charge and curvature to the membrane surface promoting electrostatic attraction and retention of proteins. It is becoming increasingly evident that lateral lipid asymmetry is of critical importance in membrane sorting during phagocytosis. Differential sorting at the plasma membrane predisposes the ensuing intracellular fate of an engulfed particle [19].

Phosphoinositide metabolism in particular is a key early event in phagocytosis [18]. PI(4)P-5kinase is recruited in response to FcγR ligation and activated by Rac1 leading to the conversion of membrane lipids to phosphoinositide PI(4,5)P_2_ in the phagocytic cup. Subsequently, PI(4,5)P_2_ recruits proteins necessary for actin assembly. PI3K is activated by the engaged FcγRand phosphorylates PI(4,5)P_2_ to PI(3,4,5)P_3_, which accumulates at the phagocytic cup and allows for phagocytic cup closure and phagosome internalization [20]. PI(3,4,5)P_3_ is an important second messenger recruiting Pleckstrin Homology domain-containing signaling proteins like Gab2 [21], Vav [22] and Akt/PKB [23]. Subsequent hydrolysis of PI(3,4,5)P_3_ to PI(3,4) P_2_ by the inositol phosphatase SHIP-1 could turn off the PI(3,4,5)P_3_ dependent signals and promote later phagosomal stages [24].

The anti-inflammatory cytokine IL-4 exerts its effects by signaling through the type I and II IL-4 receptors [25]. Interestingly, triggering of type I IL-4 receptors induces phosphorylation of IRS1/2 leading to the activation of PI3K, a pathway that is linked to the induction of genes associated with alternatively activated MΦs. Considering the involvement of PI3K in both the FcγR and IL-4 signaling pathways, we hypothesized that phagocytosis in the presence of IL-4 influences PI3K activity, thereby shifting the balance in phosphoinositide conversion and potentially altering the phagosome phenotype.

Here, we investigated in MΦs the effect of direct IL-4 signaling on lipid remodeling at the onset of phagocytosis of IgG-opsonized zymosan, a well established model microbial particle [26]. By combining biochemical methods with time-lapse confocal microscopy of GFP-tagged lipid binding probes, we monitored PI3K activity as well as the dynamic conversion and distribution of phosphoinositide species in real time during phagocytosis. We demonstrate that the presence of IL-4 induces a prolonged negative surface potential at the phagosomal membrane, leading to a prolonged recruitment of different phagosome associated proteins and subsequent changes in phagosome maturation. Our results demonstrate that phagocytosis can be modulated by cytokines secreted into the extracellular environment, most likely through cross-talk between membrane-bound uptake and signaling receptors such as the Fcγ receptors and IL-4 receptors. Furthermore, the effects of the direct IL-4 signaling at the onset of phagocytosis reported here lead to a different phagosomal phenotype that could initiate the alternative activation. The insight gained in the IL-4 induced signaling processes will provide relevant information for IL-4 related therapeutic treatments of allergic, infectious and auto-immune diseases.

## Results

### IL-4 prolongs the presence of anionic lipids on the phagosome

Previous research in MΦs showed that the surface potential of the cell membrane inner leaflet decreased locally during phagosome formation [27], and this change was attributed to depletion of anionic lipids. Prolonged exposure of MΦs to IL-4 has been shown to affect their phagocytic capacity [28], however no information is available about the direct effects of IL-4 on phagocytosis at the molecular level.

**Figure 1:**
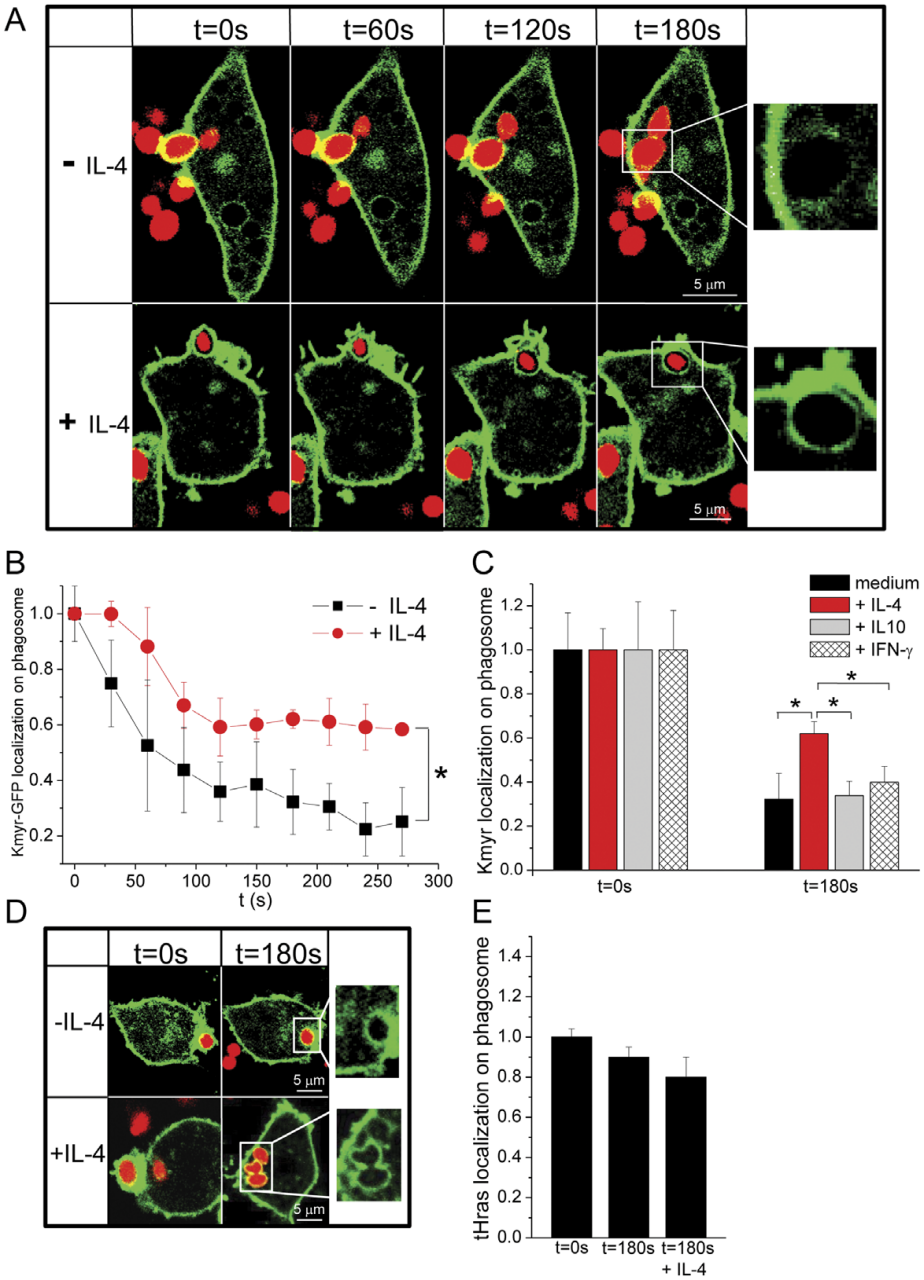
IL-4 prolonges the localization of anionic lipids during the phagocytic uptake of IgG opsonized zymosan. (A) Serum-starved RAW MΦs stably expressing Kmyr-GFP (green) were incubated or not with IL-4 (10 ng/ml) for 1 hr at 37°C prior addition of Alexa633-labelled IgG-opsonized zymosan (red) at a ratio of 1∶10, respectively. Internalization of zymosan by the MΦs was monitored over time (time lag 30 sec) by 3D confocal microscopy. The images were chosen from the Z-stack which had the optimal focus for the center cross-section of the phagosome and are representative of several similar experiments (N = 12 and N = 17 from 5 independent experiments in the absence and presence of IL-4 resp.). Scale bar indicates 5 µm. (B) The fluorescence intensity of Kmyr-GFP on the phagosomal membrane was quantified over time. To adjust for differences in Kmyr expression levels among different cells, the fluorescence intensity at the phagosomal membrane was normalized to the mean fluorescence signal at the plasma membrane for each time point, and plotted after subsequent normalization to t0. The values at each time point represent the average +/− SD obtained from phagosomes in MΦs that were untreated (black squares, N = 12) or shortly exposed (1 hr) to IL-4 (10 ng/ml) (red circles, N = 17). * indicates P<0.005 as determined by student T test. (C) Serum-starved MΦs stably expressing Kmyr-GFP were preincubated in medium alone (N = 12) or with IL-4 (10 ng/ml) (N = 17), or IL-10 (10 ng/ml) (N = 7) or IFN-γ (10 ng/ml) (N = 8) for 1 hr at 37°C. Subsequently, Alexa633-labelled IgG-opsonized zymosan was added (MΦs∶zymosan ratio was 1∶10) and phagocytosis was monitored by 3D confocal microscopy. The mean fluorescence intensity of Kmyr-GFP +/− SD on the phagosomal membranes was determined at t0 (phagosomal cup closure) and after 180 sec and normalized as indicated in B. * indicates P<0.005 as determined by student T test. (D) Serum-starved RAW MΦs stably expressing tHRas-GFP (green) were incubated or not with IL-4 (10 ng/ml) for 1 hr at 37°C prior addition of Alexa633-labelled IgG-opsonized zymosan (red) at a ratio of 1∶10, respectively. Internalization of zymosan by the MΦs was monitored over time (time lag 30 sec) by 3D confocal microscopy. The images were chosen as described in A. Scale bar indicates 5 µm. (E) The mean fluorescence intensity of tHras-GFP +/− SD on the phagosomal membranes was determined at t0 (phagosomal cup closure) and after 180 sec (with (N = 4) and without (N = 4) preincubation in IL-4) and normalized as indicated in B. All data were obtained from >3 independent experiments.

To determine whether IL-4 affects the lipid composition of the phagosomal membrane, we investigated lipid remodeling during phagocytosis of IgG-opsonized zymosan particles in the presence of IL-4. We examined the effect of IL-4 on the charge of the phagosome membrane by monitoring the recruitment of the GFP-tagged polycationic probe Kmyr, a K-ras-derived peptide that binds to anionic lipids [27], to the phagosomes. Serum-starved MΦs stably expressing Kmyr-GFP were incubated with fluorescent opsonized zymosan particles, and phagocytosis was monitored by time-lapse confocal microscopy. Internalization of the zymosan particle was confirmed by imaging in 3D in time. The results were compared with phagocytosing MΦs that were pre-incubated with IL-4 for 1 hr at 37°C (Fig. 1). In the absence of IL-4, Kmyr-GFP was localized on the phagosomal cup membrane, in agreement with published observations [27]. As expected, upon sealing of the phagosome, a gradual decrease of Kmyr from the internalized phagocytic vesicle was observed (Fig. 1A). At 180 s after phagocytic cup closure, there was only a ∼30% Kmyr-GFP residual on the phagosomal membrane as compared to the plasma membrane (Fig. 1B). In the presence of IL-4, zymosan particles were also taken up by MΦs but the localization of Kmyr-GFP at the phagosomal membrane was significantly prolonged with Kmyr localization stabilized at ∼60% for up to 300 s after phagocytic cup closure (Fig. 1A, B). In the presence of IL-4, Kmyr did eventually subside from the phagosomal membrane, but on a much longer timescale (5–10 min, data not shown) as compared to the phagosomes formed in the absence of IL-4. It should be noted that to adjust for differences in Kmyr-GFP expression levels between cells, the signal of Kmyr at the phagosome was normalized to the Kmyr signal at the plasma membrane (see Figure S1). Furthermore, flow cytometry experiments confirmed that in the absence of phagocytosis, 1 hr IL-4 treatment did not alter the expression level of Kmyr-GFP nor its predominantly uniform localization at the plasma membrane (Figure S2).

The difference in Kmyr localization was further quantified in fixed cell experiments. After synchronizing phagocytosis, cells activated or not with IL-4 were quickly fixed and the percent of phagosomes containing Kmyr was determined (Figure S3A,B). Consistent with the live cell imaging results, IL-4 prolonged Kmyr residency on the phagocytic membrane. In the presence of IL-4, 74% of the phagosomes maintained a Kmyr ring compared to only 14% in the absence of IL-4. Longer incubation times (48 hr) with IL-4 showed similar results with 78% of phagosomes maintaining a Kmyr ring (Figure S3C). The IL-4 induced differences were observed at a concentration of 10 ng/ml and higher (Figure S3D). As expected, phagocytosis of the non-opsonized zymosan particles was very low compared to IgG-opsonized zymosan (Figure S3E). Furthermore, IL-4 had no significant effect on the phagocytic capacity (Figure S3E). The IL-4 induced differences were due to early signaling events and not due to IL-4 induced upregulation or downregulation of genes, since washing out IL-4 after 1 hr recovered the distribution pattern of Kmyr during phagocytosis (Figure S3D).

We next confirmed that the effect on Kmyr distribution during phagocytosis of IgG-opsonized zymosan was IL-4 specific. First, heat-inactivated IL-4 did not induce any prolonged localization of Kmyr on the phagosomes (Figure S3D). Moreover, IL-10, which is also an anti-inflammatory cytokine but induces different effects than IL-4 on MΦ function [6], had no significant effect on Kmyr distribution (Fig. 1C). Similarly, IFN-γ, which induces the classically defined MΦ activation, did not modulate the presence of Kmyr on the phagosome (Fig. 1C).

Lipid remodeling during phagocytosis was also investigated with the lipid raft probe tHras, which binds to the plasma membrane inner leaflet indepent from the lipid charge [27]. In agreement with previous observations, tHras-GFP was localized on the membrane during the formation of the phagocytic cup and only decreased slightly over time (Fig. 1D). Unlike Kmyr-GFP, the localization of tHras-GFP on the phagosomal membrane was not significantly different in the absence and presence of IL-4 after 180 s from the phagocytic cup closure (Fig. 1D, E). tHras is a marker for lipid rafts and shows a differential subcellular compartmentalization with respect to Kmyr [29], suggesting that the tHras domains are not affected by IL-4 signaling, at least during phagocytosis.

We have shown that IL-4 activation induced a prolonged localization of the polycationic probe Kmyr on the phagosomal membrane. These results demonstrate that activation of MΦs via IL-4 affects lipid remodeling of the phagosomal membrane by prolonging the presence of anionic lipid species thereby changing the surface potential of the outer leaflet of the phagosomal membrane.

### IL-4 changes phosphoinositide composition on the phagosome

**Figure 2:**
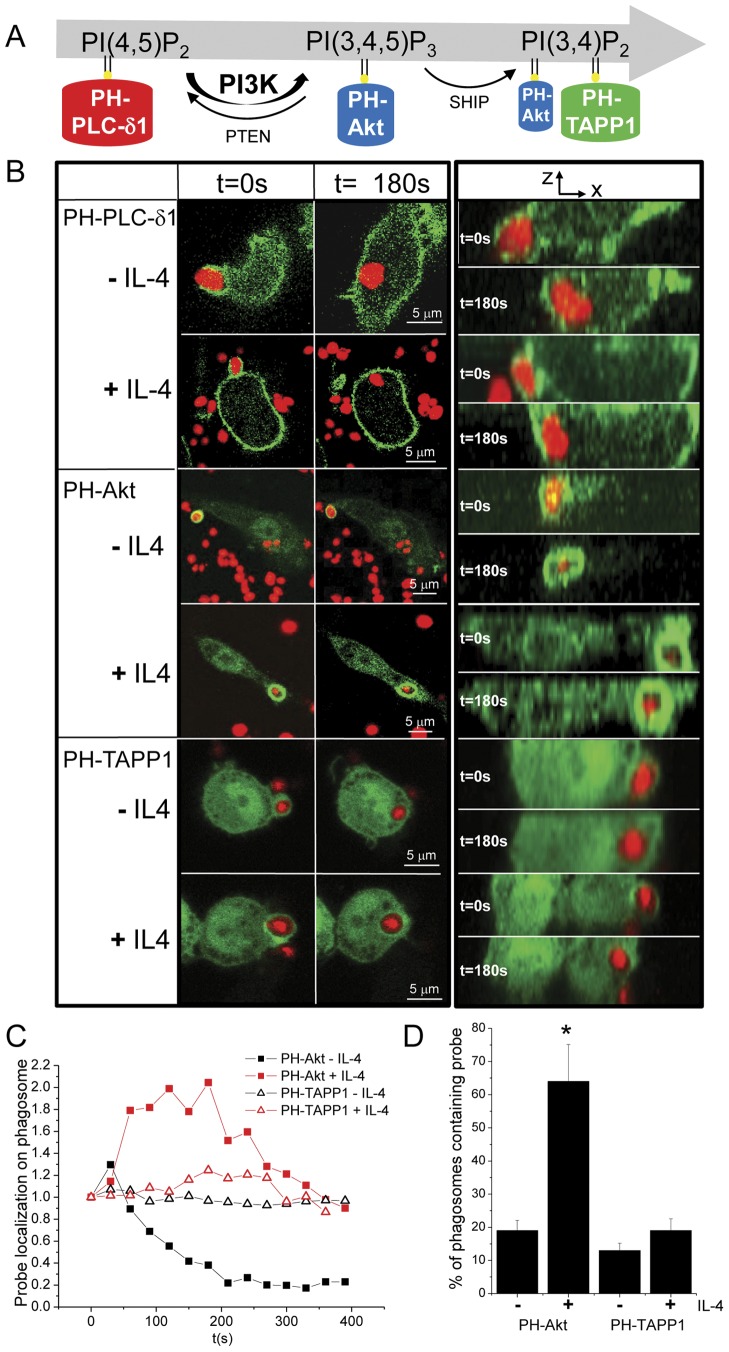
Short-term exposure to IL-4 increases PI(3,4,5)P3 levels at the phagosomal membrane. (A) Phosphoinositides are converted during phagocytosis and the different phosphoinositides can be visualized by fluorescently labeled PH-domains from specific proteins. This cartoon depicts the GFP-tagged probes used. (B) Serum-starved RAW MΦs transiently expressing the lipid binding probes PH-PLCδ- GFP, or PH-Akt-GFP or PH-TAPP1-GFP (green) were incubated or not with IL-4 (10 ng/ml) for 1 hr at 37°C prior addition of Alexa633-labelled IgG-opsonized zymosan (red) at a ratio of 1∶10, respectively. Internalization of zymosan by the MΦs was monitored over time (time lag 30 sec) by 3D confocal microscopy. The left images show the optimal focus for the center cross-section of the phagosome from the Z-stack at t = 0 s and t = 180 s and are representative of data obtained in several experiments (N = 5 for each lipid binding probe in both absence and presence of IL-4 obtained from >3 independent experiments). The right images are the orthoganol projections. Scale bar indicates 5 µm. (C) The fluorescence intensity of PH-Akt-GFP and PH-TAPP1-GFP on the phagosomal membrane was quantified over time and plotted after subsequent normalization to t0. (D) Serum starved MΦs transient expressing PH-Akt-GFP and PH-TAPP1-GFP were stimulated or not with IL-4 (10 ng/ml) at 37°C and subsequently challenged with Alexa633-labelled IgG-opsonized zymosan (1∶10 ratio) at room temperature (at which temperature no phagocytosis occurs) for 30 min after which they were shifted to 37°C to synchronize phagocytosis. After 10 min at 37°C, the cells were quickly fixed in 4% PFA, mounted in anti-fading reagent, and PH-Akt-GFP and PH-TAPP1-GFP distribution on the phagosome was analyzed by 3D confocal laser scanning microscopy. The number of PH-Akt-GFP and PH-TAPP1-GFP bearing phagosomes was determined as the fraction of total observed phagosomes (N = 90 from 3 independent experiments) ± SE. * indicates P<0.005 as determined by Fisher's exact test.

Phosphoinositides in the inner leaflet of the plasma membrane are anionic lipids and recruit pleckstrin homology (PH) domain-containing proteins that then drive further signaling. The conversion of phosphoinositides during FcγR-mediated phagocytosis possibly coordinate the different stages of phagocytosis [12]. In order to test whether the cytokine IL4 prolongs the negative charge of the phagosomal membrane by altering the conversion of phosphoinositides, we used GFP fusion constructs of PH-domains of different proteins: the PLCδ PH-domain that binds to PI(4,5)P_2_ [30], the PH-domain of Akt that binds to PI(3,4,5)P_3_ and to a lesser extent to PI(3,4)P_2_ [31], [32] and the PH-domain of TAPP1 that binds to PI(3,4)P_2_ [33] (Fig. 2A). By time-lapse confocal microscopy, we determined the conversion of phosphoinositides during phagocytosis of IgG-opsonized zymosan by monitoring the recruitment of these GFP-tagged probes to the phagosomes in the presence or absence of IL-4. No differences were observed in PLCδ PH-domain localization on the phagosomal membrane in the absence and presence of IL-4 (Fig. 2B), indicating that the levels of PI(4,5)P_2_ were not altered by IL-4. This was further supported by using a more sensitive probe consisting of two tandem PH domains of PLCδ (2PH- PLCδ–GFP) [34], which again showed no altered levels of PI(4,5)P_2_ to the phagosome in the presence of IL-4 (data not shown). Interestingly, PH-Akt which is known to recruit to the phagosomal membrane [35], showed a prolonged phagosomal localization in the presence of IL-4, while PH-TAPP1 localization was found to be unaltered by IL-4 treatment (Fig. 2B, C). Since the extent of recruitment varied among cells, we further quantified recruitment of PH-Akt and PH-TAPP1 in fixed cell experiments (Fig. 2D). In the presence of IL-4, 64% of phagosomes formed within 10 min after induction of phagocytosis showed PH-Akt localization at their membrane compared to only 19% in the absence of IL4. Since PH-TAPP1 localization was unaltered, we propose that the difference in phosphoinositides is at the level of PI(3,4,5)P_3_, which is the product of class I PI3K activity.

PI(3,4)P_2_ and PI(3,4,5)P_3_ are the second messengers which activate molecules via binding to PH domains of the downstream target proteins. Our results show that the phosphoinositide conversion is clearly altered in the presence of IL-4 with a prolonged presence of the negatively charged second messenger PI(3,4,5)P_3_, suggesting the involvement of PI3K in the IL-4 mediated effects.

### IL-4 enhances PI3K/Akt activity

**Figure 3:**
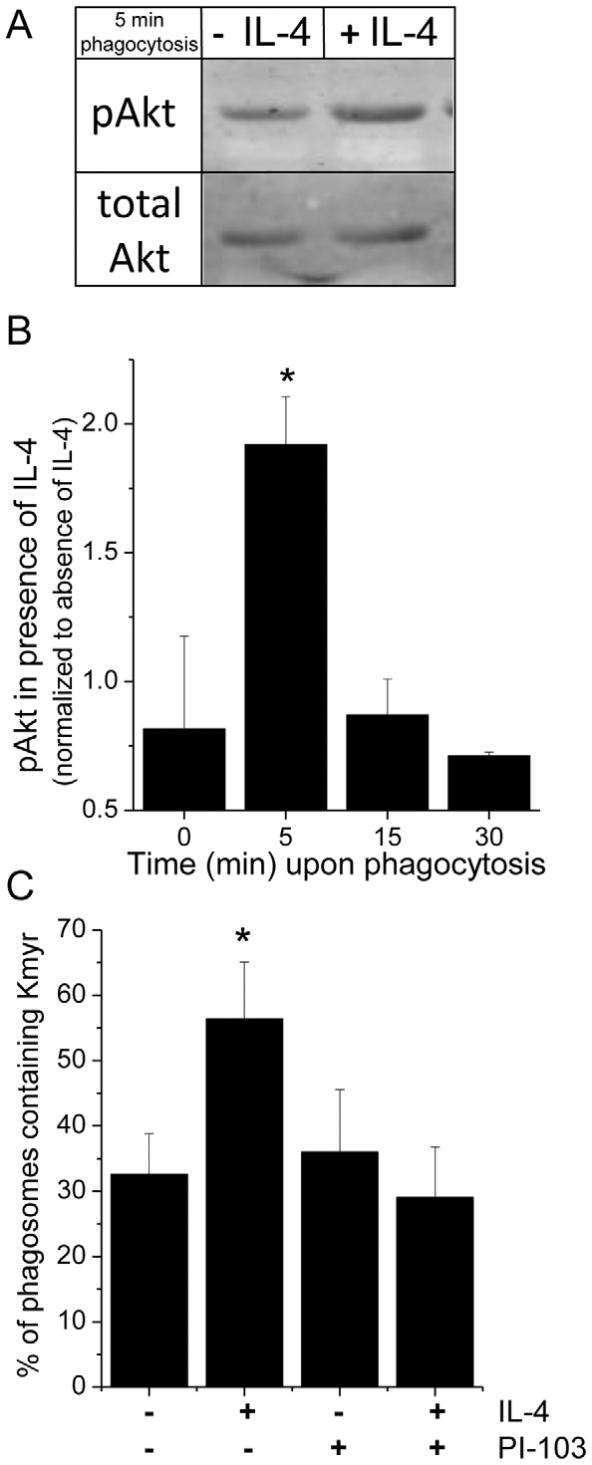
IL-4 induces increased PI3K activity during phagocytosis. (A) Serum starved Raw MΦs were stimulated or not with IL-4 (10 ng/ml) at 37°C and subsequently challenged with IgG-opsonized zymosan (1∶10 ratio) at room temperature (at which temperature no phagocytosis occurs) for 30 min after which they were shifted to 37°C to synchronize phagocytosis. The cells were lysed at different timeperiods of phagocytosis. Proteins were separated, transferred to PVDF membranes and Western blotted with a rabbit anti-phosphoserine Akt specific antibody or with a rabbit total Akt specific antibody. Shown is the immunoblot at 5 min phagocytosis and similar results were obtained in three independent experiments. (B) The fluorescence from the immunoblots was quantified using Odysey 2.1 software and the phosphospecific signal at each timepoint was normalized to the total amount of Akt in the lysate at that timepoint. The values in the presence of Il-4 (10 ng/ml, 1 hr) were plotted as a –fold increase over samples in the absence of IL-4 at each timepoint. The values represent three independent experiments. * indicates P<0.05 as determined by student T test. (C) Serum starved MΦs stably expressing Kmyr-GFP were stimulated or not with IL-4 (10 ng/ml) and subsequently challenged with Alexa633-labelled IgG-opsonized zymosan (1∶10 ratio) at room temperature (at which temperature no phagocytosis occurs) for 30 min after which they were shifted to 37°C to synchronize phagocytosis. 5 min after the temperature shift PI-103 (100 nM) was added. After 10 min at 37°C, the cells were quickly fixed in 4% PFA, mounted in anti-fading reagent, and Kmyr-GFP distribution on the phagosome was analyzed by 3D confocal laser scanning microscopy. The number of Kmyr-GFP bearing phagosomes was determined as the fraction of total observed phagosomes (N = 90 from 3 independent experiments) ± SE. * indicates P<0.05 as determined by Fisher's exact test.

The class I PI3K is responsible for the conversion of phosphoinositides by phosphorylating PI(4,5)P_2_ to PI(3,4,5)P_3_, with the latter more negatively charged. Interestingly, PI3K is also downstream of the IL-4 receptor as part of the IRS-1/2 pathway [36]. Therefore, we wanted to examine whether after short-term exposure to IL-4 an increased class I PI3K activity was responsible for the prolonged negative charge as a result of protracted localization of PI(3,4,5)P_3_ at the phagosomal membrane. We assessed serine phosphorylation of Akt, the PI3K activation reporter kinase, by Western blotting whole cell-lysates from Mφs that were or were not stimulated with IL-4. In agreement with previous work [23], Mφs that were incubated with IgG-opsonized zymosan particles for different periods of time showed an increase in Akt phosphorylation after 5 min of phagocytosis. This increase was significantly larger in the presence of IL-4 (Fig. 3A). In particular, in the presence of IL-4, a 2-fold increase in Akt phosphorylation occurred after 5 min of phagocytosis as compared to in the absence of IL-4 (Fig. 3B). The IL-4 induced increase in Akt phosphorylation was predominantly detected within the first minutes after the onset of phagocytosis, indicating the transient nature of this event in the signaling pathway. To determine whether class I PI3K activity was responsible for the prolonged negative charge and consequent prolonged Kmyr localization at the phagosomal membrane, we specifically blocked class I PI3K by adding the inhibitory drug PI-103 after closure of the phagocytic cup. PI-103 is a potent, cell-permeable, ATP-competitive inhibitor of PI3K family members with selectivity toward class I PI3K (p110α) [17]. By allowing the formation of a phagocytic cup before addition of the inhibitor, we made sure that the phagocytosis rate was unaltered (data not shown) so that the localization of Kmyr-GFP on the phagosome could be analyzed (Figure S4A). Under these conditions, the IL-4 induced prolonged Kmyr localization on the phagosome was completely abrogated (Fig. 3C).

These data demonstrate that in conjunction with phagocytosis of IgG-opsonized zymosan IL-4 increases PI3K/Akt activity which accounts for the change in phosphoinositide conversion, the extended localization of PI(3,4,5)P_3_, thereby changing the charge of the phagosomal membrane.

### IL-4 modulates the recruitment of cytoplasmic proteins to the phagosomal membrane

**Figure 4:**
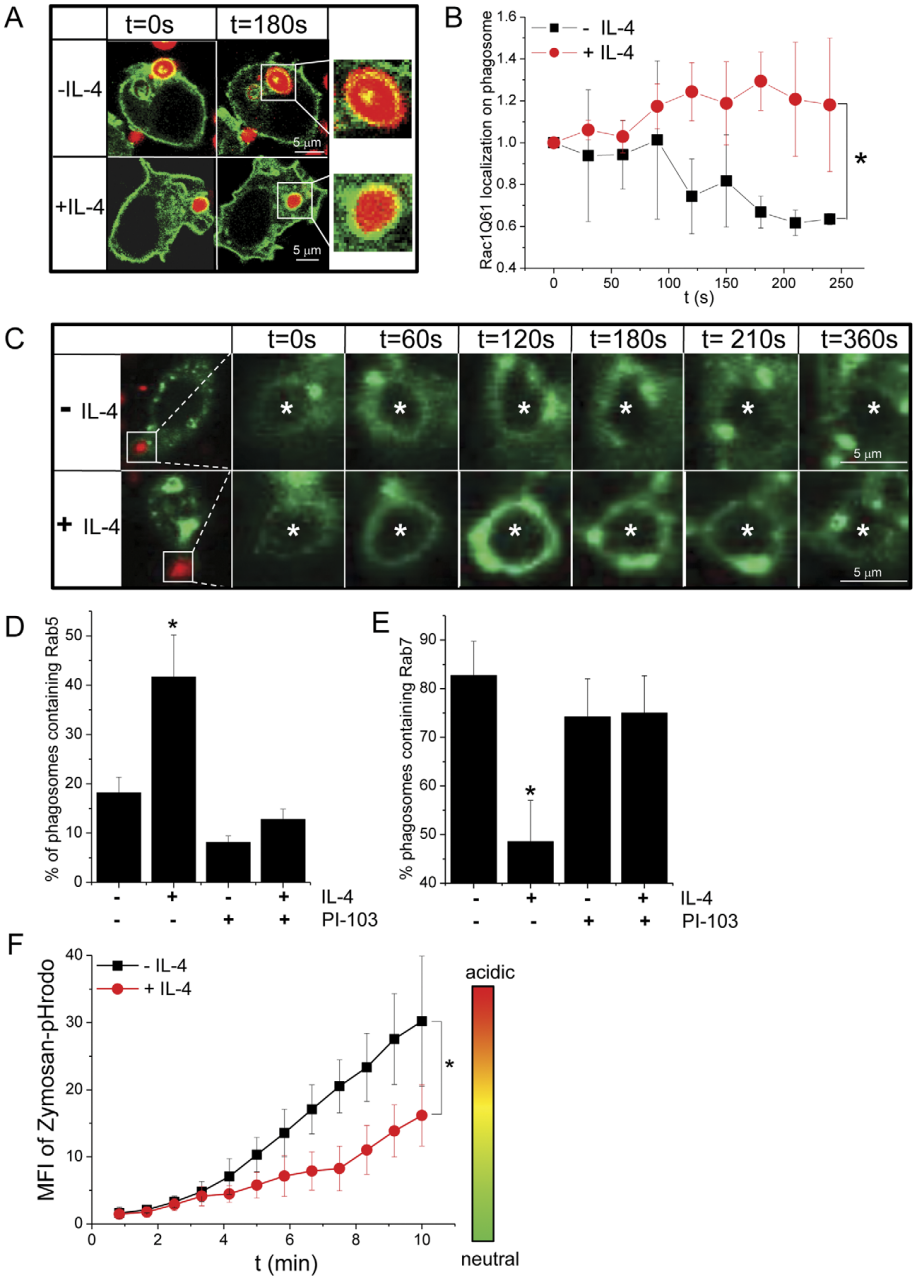
IL-4 induces altered phagosome phenotype. (A) Serum-starved RAW MΦs transiently expressing Rac1Q61-YFP (green) were incubated or not with IL-4 (10 ng/ml) for 1 hr at 37°C prior addition of Alexa633-labelled IgG-opsonized zymosan (red) at a ratio of 1∶10, respectively. Internalization of zymosan by the MΦs was monitored over time (time lag 30 sec) by 3D confocal microscopy and the images show the optimal focus for the center cross-section of the phagosome from the Z-stack at t = 0 s and t = 180 s and are representative of several similar experiments. Scale bar indicates 5 µm. (B) The fluorescence intensity of Rac1Q61-YFP on the phagosomal membrane was quantified over time. To adjust for differences in Kmyr expression levels among different cells, the fluorescence intensity at the phagosomal membrane was normalized to the mean fluorescence signal at the plasma membrane for each time point, and plotted after subsequent normalization to t0. The values at each time point represent the average +/− SD obtained from several phagosomes in MΦs that were untreated (black squares, N = 5)) or shortly exposed (1 hr) to IL-4 (10 ng/ml) (red circles, N = 5). * indicates P<0.05 as determined by student T test. (C) Serum-starved RAW MΦs transiently expressing Rab5-GFP (green) were incubated or not with IL-4 (10 ng/ml) for 1 hr at 37°C prior addition of Alexa633-labelled IgG-opsonized zymosan (red) at a ratio of 1∶10, respectively. Internalization of zymosan by the MΦs was monitored over time (time lag 30 sec) by 3D confocal microscopy and the images show the optimal focus for the center cross-section of the phagosome from the Z-stack and are representative of several similar experiments (N = 2 and N = 3 in absence and presence of IL-4 resp.). Scale bar indicates 5 µm and zymosan localization is indicated with *. Serum starved MΦs transiently expressing Rab5-GFP (D) or Rab7-GFP (E) were stimulated or not with IL-4 (10 ng/ml) and subsequently challenged with Alexa633-labelled IgG-opsonized zymosan (1∶10 ratio) at room temperature (at which temperature no phagocytosis occurs) for 30 min after which they were shifted to 37°C to synchronize phagocytosis. 5 min after the temperature shift PI-103 (100 nM) was added. After 10 min at 37°C, the cells were quickly fixed in 4% PFA, mounted in anti-fading reagent, and Rab5-GFP distribution on the phagosome was analyzed by 3D confocal laser scanning microscopy. The number of Rab5-GFP (D) or Rab7-GFP (E) bearing phagosomes was determined as the fraction of total observed phagosomes (N = 90 from 3 independent experiments) ± SE. * indicates P<0.05 as determined by Fisher's exact test. (F) Serum starved MΦs were challenged with zymosan labeled with the pH-sensitive dye pHrodo, which is nonfluorescent at neutral pH and fluoresces bright red in acidic environments. Internalization of pHrodo-zymosan by the MΦs was monitored over time (time lag 50 sec) by 3D confocal microscopy and the mean fluorescence intensity of three cross-section of the phagosome from the Z-stack was calculated at each timepoint. The values at each time point represent the average +/− SD obtained from several phagosomes in MΦs that were untreated (black squares, N = 8)) or shortly exposed (1 hr) to IL-4 (10 ng/ml) (red circles, N = 6) and are representative of three independent experiments. * indicates P<0.005 as determined by student T test.

The phagosome matures by changing the molecules associated with its membrane during its route through the cell [37]. The small GTPase Rac1, which is recruited to the plasma membrane upon activation of FcγRs and is important for actin assembly during phagocytosis [38], contains a polybasic domain like K-ras and its localization to the plasma membrane is sensitive to the surface potential [39]. Since Rac1 localizes to the plasma membrane in a similar fashion as Kmyr, we investigated whether short-term exposure of MΦs to IL-4 affected the recruitment of Rac1 to the phagosome during phagocytosis of IgG-opsonized zymosan. For these experiments, MΦs were transiently transfected with constitutively active form of Rac1, Rac1(Q61L)-YFP (Fig. 4A), which binds to the plasma membrane independently from nucleotide hydrolysis or cessation of nucleotide exchange [27]. As expected, in untreated cells, Rac1(Q61L)-YFP was localized to the plasma membrane and the distribution during phagocytosis was comparable to Kmyr, with decreased levels on the phagosomal membrane after phagocytic cup closure. In contrast, the presence of IL-4 induced a prolonged localization of Rac1(Q61L)-YFP on the phagosomal membrane as also observed for Kmyr-GFP (Fig. 4A, B).

A further downstream protein in the phagosomal signaling process is the small rabGTPase Rab5. RabGTPases are thought to orchestrate the sequence of fusion events of compartments of the endocytic pathway with the phagosome during the maturation process, leading to the formation of the phagolysosome [16]. Rab5, which is recruited rapidly and transiently to the phagosome, is known to be essential for the recruitment of Rab7 and for progression to phagolysosomes [40], as the maturation from early to late phagosome is determined by the switch from Rab5 to Rab7. The localization of Rab5-GFP was followed during the uptake of IgG-opsonized zymosan in the absence and presence of IL-4 (Fig. 4C). The results show that in the presence of IL-4 there was a prolonged localization of Rab5 on the phagosome as determined by both live cell imaging and fixed cell experiments (Fig. 4C, D). Consistent with this, Rab7 showed a delayed recruitment to the phagosome as visualized with Rab7-GFP in the fixed cell experiments (Fig. 4D).

Finally, we investigated whether the IL-4 induced effect on Rab5 and Rab7 localization was the result of a cross-talk between IL4 receptor and FcγR at the level of PI3K. To this aim, PI3K was blocked rapidly after the onset of phagocytosis with the class I PI3K specific drug PI-103 and the localization of Rab5-GFP and Rab7-GFP on the phagosome was determined (Figure S4B, C). Addition of PI-103 completely abrogated the IL-4-dependent protracted localization of Rab5 and the delayed recruitment of Rab7 on the phagosome (Fig. 4D, E). These results clearly show that during phagocytosis the combined action of IL-4 receptor and FcγR on PI3K alters the levels of PI(3,4,5)P_3_ thereby changing the kinetics of the recruitment of downstream signaling molecules possibly resulting in a different phagosome phenotype.

### Phagosome acidification is delayed in short-term IL-4 activated MΦs

Phagosome maturation involves several complementary mechanisms including acidification. The prolonged residence of Rab5 at the phagosomal membrane in the presence of IL-4 suggests a difference in early phagosome phenotype that could result in an altered phagosome maturation profile. To investigate whether the short-term exposure to IL-4 induced a different phagosome fate, we determined the acidification pattern of the phagosomes formed in the absence and presence of IL-4. MΦs were exposed to opsonized zymosan particles that were coated with the pH-sensitive dye, pHrodo, which fluoresces in an acidic environment. In the presence of IL-4 the increase in fluorescence was significantly smaller for the first 10 min upon uptake as compared to untreated cells (Fig. 4E). At 10 min after the onset of phagocytosis, the phagosomal pH was ∼ between 4–5 in the absence and ∼6 in the presence of IL-4 (Figure S5). It has been reported that the acidification of phagosomes range from pH 6 up to 4.2 when phagsosomes maturate from early phagosome to phagolysosome respectively [41], [42], with the phagosomal pH acidifying to pH∼5 within the first 5 min [43]. The pH of the phagosome in the presence of IL-4 resembles that of an early phagosome (∼6) agreeing with our findings that IL-4 induced phagosomes contain the early phagosome marker Rab5. These data demonstrate that the acidification of phagosomes formed in the presence of IL-4 is decreased or delayed, indicating that changes in the phagosome phenotype modulated by extracellular factors may represent an additional mechanism that regulates the outcome of phagocytosis.

## Discussion

Lipid remodeling is an essential mechanism that regulates phagosome signaling and consequential fate. Little is known about how extracellular signals such as cytokines can influence this process. Here, we investigated the direct effect of short-term IL-4 exposure (1 hr) on lipid remodeling at the onset of phagocytosis of IgG-opsonized zymosan in MΦs. Our results show a prolonged negative charge at the membrane of early phagosomes in the presence of IL-4 and we demonstrate this is due to an extended association of the negatively charged lipid second messenger PI(3,4,5)P_3_, as a result of an increased PI3K activity. This lipid remodeling observed in the presence of IL-4 led to a significantly different early phagosome phenotype reflected by longer Rac1 and Rab5 association to the phagosomal membrane, a delayed Rab7 phagosome recruitment and phagosome acidification.

**Figure 5:**
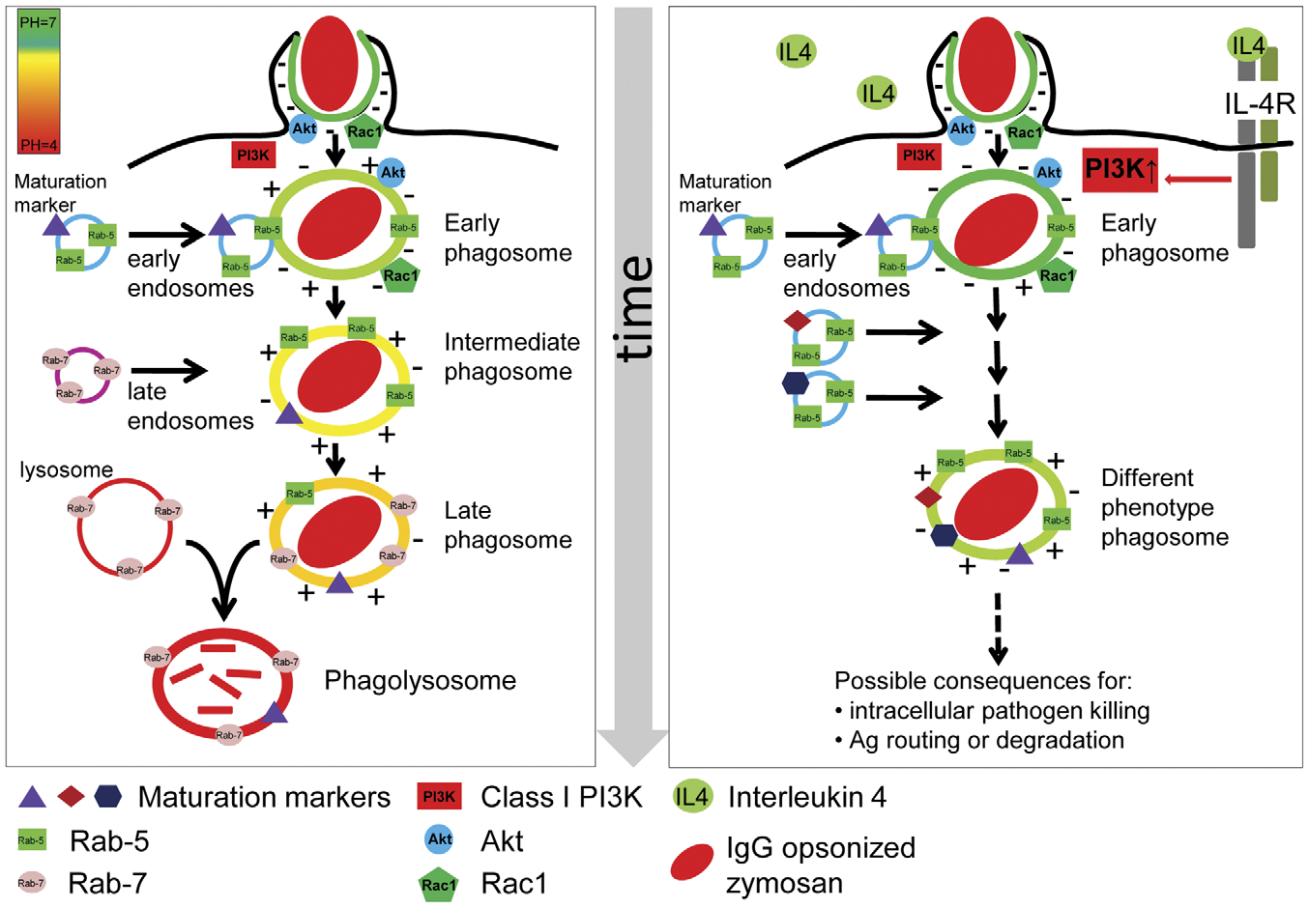
Model describing the direct effect of IL-4 on early signaling during phagocytic uptake of IgG opsonized zymosan. Our results show a prolonged negative charge at the membrane of early phagosomes in the presence of IL-4, which is the result of an extended association of the negatively charged lipid second messenger PI(3,4,5)P_3_, visualized by the prolonged localization of PH-Akt on the phagosome. We hypothesize this is the result of an increased PI3K activity due to the engagement of ITAM-domain containing receptors (FcR) in the environment of activated IL-4 receptors, which both signal downstream to PI3K. We demonstrated that indeed PI3K/Akt pathway activity is increased and blocking specifically the class I PI3K abrogates the IL-4 induced effects. IL-4 induced prolongation of PI(3,4,5)P_3_ levels can direct changes in downstream signaling in two ways. The prolonged negative membrane charge can attract cationic proteins for a longer period of time and PI(3,4,5)P_3_ itself, as an important second messenger, can signal downstream for a longer period of time. Thus the IL-4 induced change in lipid remodeling can lead to a significantly different early phagosome phenotype and we showed this by the prolonged association of Rac1 and Rab5 to the phagosomal membrane. The different phenotypic phagosome phenotype can lead to a different phagsomal fate, which we showed by a delayed phagosome acidification. This can have consequences for intracellular pathogen killing, antigen degradation and maybe even autophagy.

According to the electric-switch theory, the interaction between plasma membrane inner leaflet anionic lipids and cationic proteins can be modified by modulating either the charge of the cationic proteins or the inner leaflet potential [44]. The latter can be achieved by lipid conversion from lipids with a moderate negative charge to lipids with a high negative charge. Upon MΦ activation by short-term exposure to IL-4 during phagocytosis of IgG-opsonized zymosan, we observed a significant change in phosphoinositide content at the membrane of early phagosomes as indicated by a prolonged residence time of GFP-tagged PH-domain of Akt, which has a high affinity for PI(3,4,5)P_3_ [23]. PI(3,4,5)P_3_ is an important second messenger recruiting PH domain-containing signaling proteins like Gab2 [21], Vav [22] and Akt/PKB [23]. PH domains have now been identified in >100 different proteins, many of which are involved in regulating intracellular signaling pathways or the cytoskeleton [45]–[48]. A general consensus has emerged that PH domains function to mediate intermolecular interactions and have primarily evolved to regulate protein–lipid interactions, although in some instances PH domains may also mediate protein–protein interactions [47]–[49]. A prolonged presence of PI(3,4,5)P_3_ could induce a prolonged downstream signaling initiated by PH domain-containing proteins, with a possible influence on the phagosome fate. Furthermore, the prolonged negative charge on the phagosome membrane, as a result of PI(3,4,5)P_3_, can attract cationic proteins to the phagosomal membrane thereby changing the phagosome phenotype and/or signaling in the presence of IL-4 (Fig. 5). It has to be noted that the PH domain of Akt has been found to also bind to PI(3,4)P_2_ albeit to a lesser extent [32], [50]. However, the lack of changes in the residence time of GFP-tagged PH-domain of TAPP1, which is specific for PI(3,4)P_2_ [33], confirmed that a prolonged negative charge of the early phagosomes generated in the presence of IL-4 is due to PI(3,4,5)P_3_.

PI(3,4,5)P_3_ is the product of PI3K activity, which has been shown to be involved in both FcγR and IL-4 receptor signaling pathways [25], [51]. Indeed, the changes in lipid remodeling observed in the presence of IL-4 during FcγR engagement are a result of an increased PI3K activity, as reflected by increased phosphorylation levels of the downstream reporter protein Akt. Moreover, when class I PI3K activity was blocked by addition of a specific inhibitor (PI-103) right after phagosome sealing, the IL-4-induced increase in anionic lipids monitored by the polycationic Kmyr probe at the phagosomal membrane was abrogated. It should be noted that although we used IgG opsonized zymosan, a well-established microbial model, we cannot completely exclude the contribution of other zymosan binding receptors, other than FcγR. On MΦs, the beta-glucan receptor Dectin-1 has been shown to mediate binding and uptake of non-opsonized zymosan [52], [53]. However, similarly to FcγR, Dectin-1 also has an ITAM motif that is phosphorylated upon receptor engagement and can act as docking site for PI3K [54]. Furthermore, binding and phagocytosis of non-opsonized zymosan was negligible under our experimental conditions.

The changes in phagosome phenotype as reflected by phosphoinositide content are also observed at the protein level. Rac1 is of particular importance to FcγR mediated phagocytosis and detaches rapidly upon sealing [38]. Rac1 also contains a polybasic domain [39] and the Rac1(Q61L) mutant, which is constitutively bound to GTPand associates with the phagosomal membrane in a charge dependent manner [27], was indeed sensitive to IL-4 induced changes in membrane charge. Interestingly, Rac1 and PI3K have been reported to signal in a positive feedback loop [55], which could strengthen the sustained PI3K activity and promote PI(3,4,5)P_3_ formation. Rac1 was recently reported to control the localization of PIP5K [56], which generates PI(4,5)P_2_, the substrate of class I PI3K, during phagosome formation [57]. In the phagosomes generated via FcγR engagement in the presence of IL-4, we clearly observe extended Rac1 localization most likely due to a prolonged PIP5K localization on the phagosome membrane. Bohadowizch and colleagues recently showed that after sealing there is no PIP5K detected at the phagosomal membrane in FcγR mediated phagocytosis in contrast to Complement receptor 3 (CR3) mediated phagocytosis [17]. Our results indicate that IL-4 induces a shift in phagosome phenotype that appear to resemble a CR3 generated phagosome in terms of phosphoinositide content.

We showed that in the presence of IL-4, Rab5, a typical early phagosome marker, is retained for a longer period of time on the phagosomal membrane, while recruitment of Rab7 is delayed. Rab5 and Rab7 are known to be part of the switch that regulates early-to-late endosome transition and its displacement from the vesicle membrane is essential to promote cargo progression [58], [59]. Rab5 is important for the control of early endosome docking and fusion and its overexpression was shown to induce enlarged early endosomal compartments [60], [61]. Recently, IL-4 in combination with prostaglandin E2 was found to induce the formation of similar enlarged endosomes in a Rab5-dependent manner in mouse MΦs [7]. Our data further extend these findings by providing the molecular mechanism that explains the formation of Rab5 enriched and enlarged early phagosomes. Moreover, we can now ascribe the prolonged recruitment of Rab5 to the phagosome in the presence of IL-4 to the activity of class I PI3K.

Autophagy is a recognized immune effector mechanism against intracellular pathogens, including Mycobaterium [62]. Interestingly, it has recently been shown that short IL-4 exposure specifically restrained the transfer of Mycobacteria into lysosomes and enhanced Mycobacteria survival within infected MΦs by abolishing the protection against intracellular pathogens provided by autophagy [63]. Here, we demonstrate that the blocking effect of IL-4 on starvation-induced autophagy is dependent on signaling via the PI3K/Akt pathway.

Rab5 dependent fusion with early endosomes is required for Mycobacterium retention in early phagosomal compartments, thus promoting phagosome maturation arrest [64]. This is further supported by our observation that phagosomes generated in the presence of IL-4 showed a marked delay of acidification. Our results therefore allow us to combine these previous observations into one model that explains at the molecular level the multiple effects of the direct IL-4 signaling leading to phagosome maturation arrest and enhanced survival of intracellular pathogens like Mycobacterium.

Alternatively activated MΦs obtained via 48 hr pretreatment with IL-4 show a remarkable switch from efficient phagocytosis to reduced uptake leading to potentiated microbial-induced signaling and cytokine production, which correlated with inhibition of Akt phosphorylation [8]. After such a prolonged IL-4 treatment it is difficult to ascribe changes in phagocytosis to direct IL-4 signaling or rather to signaling as a results of IL-4 induced gene expression. In the present work, we specifically address the effects of short-term exposure (1 hr) of MΦs to IL-4 on signaling during phagocytosis and demonstrate a clear IL-4 dependent increase in PI3K/Akt activity. Although more investigation is needed, our data suggest that even a transient increase of extracellular IL-4 levels could affect the destiny of pathogens phagocytosed by macrophages.

In summary, our findings revealed novel insight into the modulating effects of extracellular factors such as IL-4 on the coordination of lipid remodeling and protein recruitment during phagocytosis. Engagement of distinct phagocytic receptors can lead to phagosomes with differing phenotypes [17]. Here, we demonstrate that engagement of the same phagocytic receptor repertoire but in different extracellular microenvironments represents an additional mechanism to regulate phagosome phenotype and destiny. In view of the upcoming model where endosomal and/or phagosomal membranes are part of the cellular network of signaling circuits by providing topological constraints to signaling molecules [65], changes in the phagosome phenotype modulated by extracellular factors may have significant impact on the net biochemical output of a cell. This highlights the importance of understanding the cooperativity between different signaling pathways during phagocytosis as this may have important consequences for the resolution of infectious diseases. Considering the emerging importance of autophagy as anti-microbial mechanism, it will be interesting to investigate the lipid remodeling and subsequent protein recruitment occurring at the membrane of autophagosomes.

## Materials and Methods

### Cell culture

Raw 246.7 cells were cultured in RPMI-1640 medium (Gibco) supplemented with 10% fetal bovine serum (FBS, Greiner Bio-one), 1 mM Ultra-glutamine (BioWitthaker) and antibiotics (100 U/ml penicillin, 100 µg/ml streptomycin and 0.25 µg/ml amphotericin B, Gibco) in a humidified, 5% CO2 containing atmosphere. Stable cell lines expressing Kmyr-GFP and tHras-GFP were maintained using the appropriate antibiotics. Transient transfections with Rab5-GFP, Rab7-GFP, RacQ61-YFP, PH-TAPP1-GFP, PH-Akt-GFP and PH-PLCδ-GFP were performed with Fugene HD according to the manufacturer protocol and imaged after 24 hrs (Roche). Cells were plated one day prior to measuring or transfection in Wilco dishes (Wilco dishes BV) at 400.000 cells/dish. Serum starvation was performed one hour before measuring by replacing the medium with 1 ml serum-free RPMI medium without phenolred to avoid autofluorescence. The cytokine-treated cells were incubated with cytokines for 1 hr and in the case of IL-4 also for 48 hr. The 48 hr IL-4 incubation was done in the presence of serum and the cells were starved one hour before measuring while the cytokine remained present in the medium.

### Labeling and opsonization of zymosan

Zymosan (Sigma) was dissolved at a concentration of 2×10^8^ particles/ml in 100 um/ml Alexa 633 carboxylic acid, succinimidyl ester (Invitrogen) in 0.05 M NaHCO_3_/Na_2_CO_3_ (pH 9.5).

After 30 min incubation, the labeled zymosan was washed with PBS + 1% BSA (PBA) buffer and stored at a concentration of 50×10^6^ particles/ml in PBA at 4C. A similar labeling was used for the pHrodo dye, also a succinimidyl ester (Invitrogen). For each experiment 50 µl of labeled zymosan particles (50×10^6^ particles/ml) were freshly opsonized by incubation for 30 min with 3 µl of mIgG (10 mg/ml) or hIgG (10 mg/ml). The particles were washed twice in PBS and suspended in serum-free RPMI.

### Cell stimulation, lysis and Western Blotting

Raw 264.7 cells prestimulated with IL-4 (10 ng/ml, 1 hr) or not were incubated with IgG opsonized zymosan at room temperature for 30 min to allow binding but not internalization; this was confirmed by confocal microscopy. In order to activate, the cells were shifted to 37°C and the medium was replaced with pre-warmed 37°C medium (including 10 ng/ml IL-4 if cells were IL-4 pretreated) to induce phagocytosis. After different time periods of phagocytosis, the cells were lysed in lysisbuffer (10 mM Tris, 150 mM NaCl, 2 mM MgCl_2_, 10% Triton X-100, 1.25 mM NaF, 0.01 mM NaPyroP_2_, 0.1 µm Na_3_VO_4_, 0.05 PMSF, 1% Complete Mini (Roche)) and lysates were boiled in SDS buffer with β-mercaptoethanol for 5 min. Proteins were separated by Western Blotting, transferred to PVDF membranes, probed with either Akt antibody (#9272, Cell signaling) or phospho Akt Ser473 193H12 antibody (#4058 Cell signaling) and a secondary Goat anti Rabbit AF680 antibody (A21076, Invitrogen) and imaged with fluorescence scanner (Odyssey 2.1). The fluorescence was quantified using Odyssey 2.1 software and the phosphospecific signal was normalized to the total amount of Akt in the lysate and the values were plotted as a –fold increase over samples that were not shifted to 37°C.

### Fixed cell imaging

The cells were incubated with IgG opsonized zymosan at room temperature for 30 min to allow binding but no internalization. The cells were shifted to 37°C and the medium was replaced with pre-warmed 37°C medium (including 10 ng/ml IL-4 if cells were IL-4 pretreated) to induce phagocytosis. The PI3K inhibitor PI-103 (Cayman Chemicals) was added after 5 min incubation at a final concentration of 100 nM. The samples were incubated for 10 min total and immediately fixed in warm 4% paraformaldehyde in PBS for 15 mins at room temperature. The samples were mounted in Mowiol and imaged using a Zeiss LSM 510 microscope equipped with a PlanApochromatic 63×/1.4 NA oil immersion objective. The samples were excited with 488 nm (GFP) Argon laser and 633 nm (Alexa 647) HeNe laser. For each sample, 20 images of cells containing phagocytosed zymosan, were taken. For every phagosome it was determined whether a ring of the GFP-tagged protein was visible around the zymosan particle. The standard error of the fraction of GFP-tagged containing phagosomes was calculated by SE = sqrt(p*(1−p)/n) with p = fraction of phagosomes with ring and n = total number of phagosomes. P-values were determined using a Fisher's exact test, to test whether the ratio of positive phagsomes to total number of measured phagsomes is significantly different with and without IL-4. The differences were considered statistically significant when p≤0.05.

### Live cell imaging

The distribution of the GFP-labeled lipid sensors, Rab5-GFP and RacQ61-YFP during uptake of IgG opsonized zymosan-Alexa633 were imaged with a confocal microscope (Olympus and Zeiss) at 37°C. The samples were excited with 488 nm (GFP) and 514 nm (YFP) Argon laser and 635 nm diode laser or 633 nm HeNe laser (Alexa 647) (Olympus and Zeiss resp.) Dual-color images were acquired every 30 s as a z-stack (1 µm) of 15 images. For every time point, the image was chosen from the z-stack which had the optimal focus for the center cross-section of the phagosome. Using ImageJ, the mean intensity/pixel was measured for the plasma membrane compartment and the phagosomal membrane compartment at each time point. Because the phagocytic cup was membrane rich (i.e. two membranes folded around a thin layer of cytoplasm), membrane-associated fluorescently labeled molecules could have seemed to be recruited to the site of phagocytosis simply as a result of the increase in membrane density. For this reason we measured the intensity of the plasma and phagosomal membranes from the point that both membranes were distinguishable. Since this coincided with the closure of the phagocytic cup, we denote the closure as time t = 0 s. First, the mean intensity/pixel of the cytosol was subtracted from these values. Then the ratio was taken by dividing the phagosomal membrane with plasma membrane, normalized to t = 0 and plotted against time (Figure S3). For probes that were not constitutively recruited to the plasma membrane (PH-Akt-GFP and PH-TAPP1-GFP) the mean intensity per pixel of the phaghosomal membrane was normalized to the mean intensity per pixel in the cytosol. P-values were determined using a student t-test, differences were considered statistically significant when p≤0.05.

The pH-sensitive dye, pHrodo, is nonfluorescent at neutral pH and fluoresces bright red in acidic environments. The fluorescent intensity of pHrodo-labeled zymosan was measured at 3 different planes every 50 s upon closure of the phacocytotic cup. The average of these three planes per particle was taken, the MFI (mean fluorescence intensity) per particle, and plotted against time.

## Supporting Information

Figure S1
**Analysis of probe localization on the phagosmal membrane.** The mean intensity/pixel was measured for the plasma membrane compartment and the phagosomal membrane compartment. Because the phagocytic cup was membrane rich (i.e. two membranes folded around a thin layer of cytoplasm), membrane-associated fluorescently labeled molecules could have seemed to be recruited to the site of phagocytosis simply as a result of the increase in membrane density. For this reason we measured the intensity of the plasma and phagosomal membranes from the point that both membranes were distinguishable. The localization of the probe on the phagosomal membrane at each timepoint was calculated with:
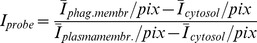
After calculation of the probe localization on the phagosomal membrane at each timepoint, the values were normalized to t0. The localization of a probe that resides in the cytoplasm (like PH-Akt) and is recruited to the phagosomal membrane was calculated with:
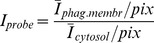

(TIF)

Figure S2 **IL-4 does not change Kmyr distribution on the plasma membrane.** In the absence of phagocytosis, MΦs showed a uniform plasma membrane localization of Kmyr-GFP both in the absence (**A**) and the presence (**B**) of IL-4 (10 ng/ml, 1 hr). The images show the optimal focus for the center cross-section of the phagosome from the Z-stack. (**C**) The expression levels of Kmyr-GFP in stably transfected MΦs before and after 1 hr IL-4 activation were determined by measuring the mean fluorescence intensity of cells. Data shown represents the average of >10 cells ± SD.
(TIF)

Figure S3 **IL-4 effect on Kmyr distribution during phagocytosis.** Serum starved MΦs stably expressing Kmyr-GFP were stimulated or not with IL-4 (10 ng/ml) and subsequently challenged with Alexa633-labelled IgG-opsonized zymosan (1∶10 ratio) at room temperature (at which temperature no phagocytosis occurs) for 30 min after which they were shifted to 37°C to synchronize phagocytosis. After 10 min at 37°C, the cells were quickly fixed in 4% PFA, mounted in anti-fading reagent. This time point was experimentally chosen to provide the optimal amount of early phagosomes in which we could compare the distribution of Kmyr in the absence and precence of IL-4. Kmyr-GFP distribution on the phagosome was analyzed by 3D confocal laser scanning microscopy to confirm internalization of the zymosan particle. The images are representative examples of untreated MΦs (A) or MΦs shortly exposed (1 hr) to IL-4 (10 ng/ml) (B) and were chosen from the Z-stack which had the optimal focus for the center cross-section of the phagosome. The position of the zymosan particle is indicated with *. The integrated fluorescence intensity values along the rectangle (10 pixels wide) crossing the cell in the image is plotted. Scale bar indicates 5 µm. (C) The number of Kmyr-GFP bearing phagosomes was determined as the fraction of total observed phagosomes ± SE in untreated or 1 hr IL-4 treated or 48 hrs IL-4 treated cells (* p<0.005 as determined by Fisher's exact test). (D) The number of Kmyr-GFP bearing phagosomes was determined as the fraction of total observed phagosomes ± SE in untreated cells or cells treated with either 1 ng/ml, 10 ng/ml and 100 ng/ml IL-4 (1 hr) (* p<0.005 as determined by Fisher's exact test). Kmyr-GFP bearing phagosomes were also monitored after washing away the IL-4 after 1 hr treatment (10 ng/ml) and allowing the cells recover for 1 hr, and upon treatment with heat-inactivated IL-4 (10 ng/ml, 1 hr). (E) The number of phagocytosis events of IgG-opsonized zymosan and non-opsonized zymosan was determined in the absence and presence of IL-4 (*p<0.05 as determined by Fisher's exact test). Data shown represents the fraction ± SE in three independent experiments and the total observed phagosomes was >30 in each experiment.
(TIF)

Figure S4 **Blocking class I PI3K abrogates the IL-4 induced changes during phagocytosis.** Serum starved MΦs stably expressing Kmyr-GFP (A), transiently expressing Rab5-GFP (B) or Rab7-GFP (C) were stimulated or not with IL-4 (10 ng/ml) and subsequently challenged with Alexa633-labelled IgG-opsonized zymosan (1∶10 ratio) at room temperature (at which temperature no phagocytosis occurs) for 30 min after which they were shifted to 37°C to synchronize phagocytosis. 5 min after the temperature shift PI-103 (100 nM), a specific class I PI3K inhibitor, was added. After 10 min at 37°C, the cells were quickly fixed in 4% PFA, mounted in anti-fading reagent, and Kmyr-GFP or Rab5-GFP distribution on the phagosome was analyzed by 3D confocal laser scanning microscopy. Scale bars indicate 3 µm.
(TIF)

Figure S5 **pH range of zymosan labeled with pHrodo.** Zymosan particles labeled with the pH-sensitive dye pHrodo, which is nonfluorescent at neutral pH and fluoresces bright red in acidic environments, were placed on Poly-L Lysine coated Wilco dishes (Wilco dishes BV). Fluorescence of the same pHrodo-zymosan particles was monitored at different pH by 3D confocal microscopy and the mean fluorescence intensity of three cross-section of the pHrodo-zymosan particle from the Z-stack was calculated at each timepoint. The values at each pH represent the average +/− SD obtained from multiple pHrodo-zymosan particle (N = 30). The data were compared with the MFI obtained for pHrodo-zymosan containing phagosomes in MΦs untreated or shortly exposed (1 hr) to IL-4 (10 ng/ml) 10 min upon phagocytosis.
(TIF)
