# Supplementary figures and images for: Interleukin-4 Alters Early Phagosome Phenotype by Modulating Class I PI3K Dependent Lipid Remodeling and Protein Recruitment
Source: PLoS One. 2011 Jul 25;6(7):e22328. doi: 10.1371/journal.pone.0022328 (PMC3143135; doi:10.1371/journal.pone.0022328)

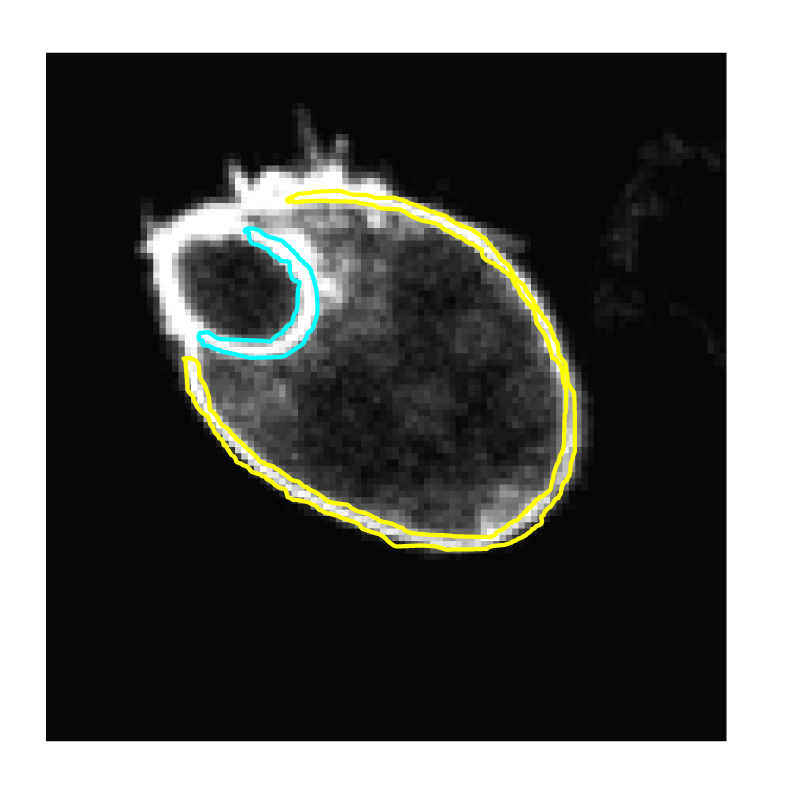

Supplement: Figure S1 — Analysis of probe localization on the phagosmal membrane. The mean intensity/pixel was measured for the plasma membrane compartment and the phagosomal membrane compartment. Because the phagocytic cup was membrane rich (i.e. two membranes folded around a thin layer of cytoplasm), membrane-associated fluorescently labeled molecules could have seemed to be recruited to the site of phagocytosis simply as a result of the increase in membrane density. For this reason we measured the intensity of the plasma and phagosomal membranes from the point that both membranes were distinguishable. The localization of the probe on the phagosomal membrane at each timepoint was calculated with:After calculation of the probe localization on the phagosomal membrane at each timepoint, the values were normalized to t0. The localization of a probe that resides in the cytoplasm (like PH-Akt) and is recruited to the phagosomal membrane was calculated with: (TIF) [file pone.0022328.s001.tif]

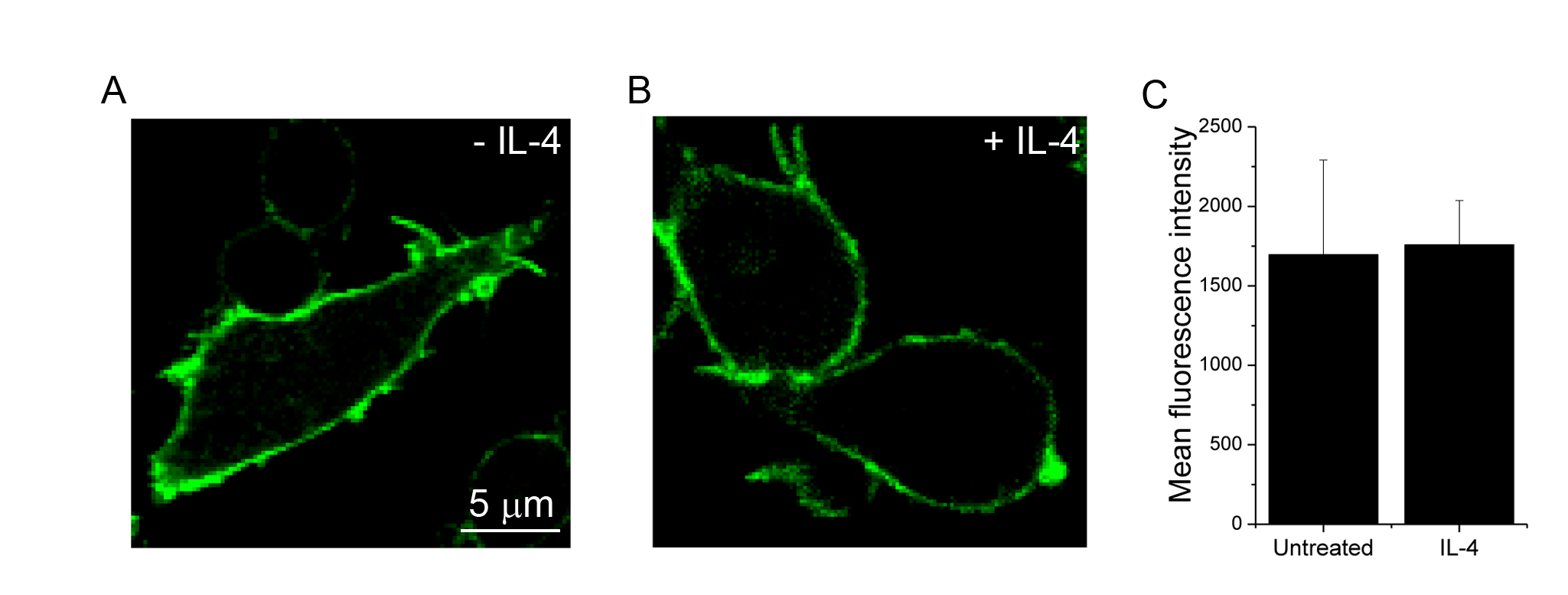

Supplement: Figure S2 — IL-4 does not change Kmyr distribution on the plasma membrane. In the absence of phagocytosis, MΦs showed a uniform plasma membrane localization of Kmyr-GFP both in the absence (A) and the presence (B) of IL-4 (10 ng/ml, 1 hr). The images show the optimal focus for the center cross-section of the phagosome from the Z-stack. (C) The expression levels of Kmyr-GFP in stably transfected MΦs before and after 1 hr IL-4 activation were determined by measuring the mean fluorescence intensity of cells. Data shown represents the average of >10 cells ± SD. (TIF) [file pone.0022328.s002.tif]

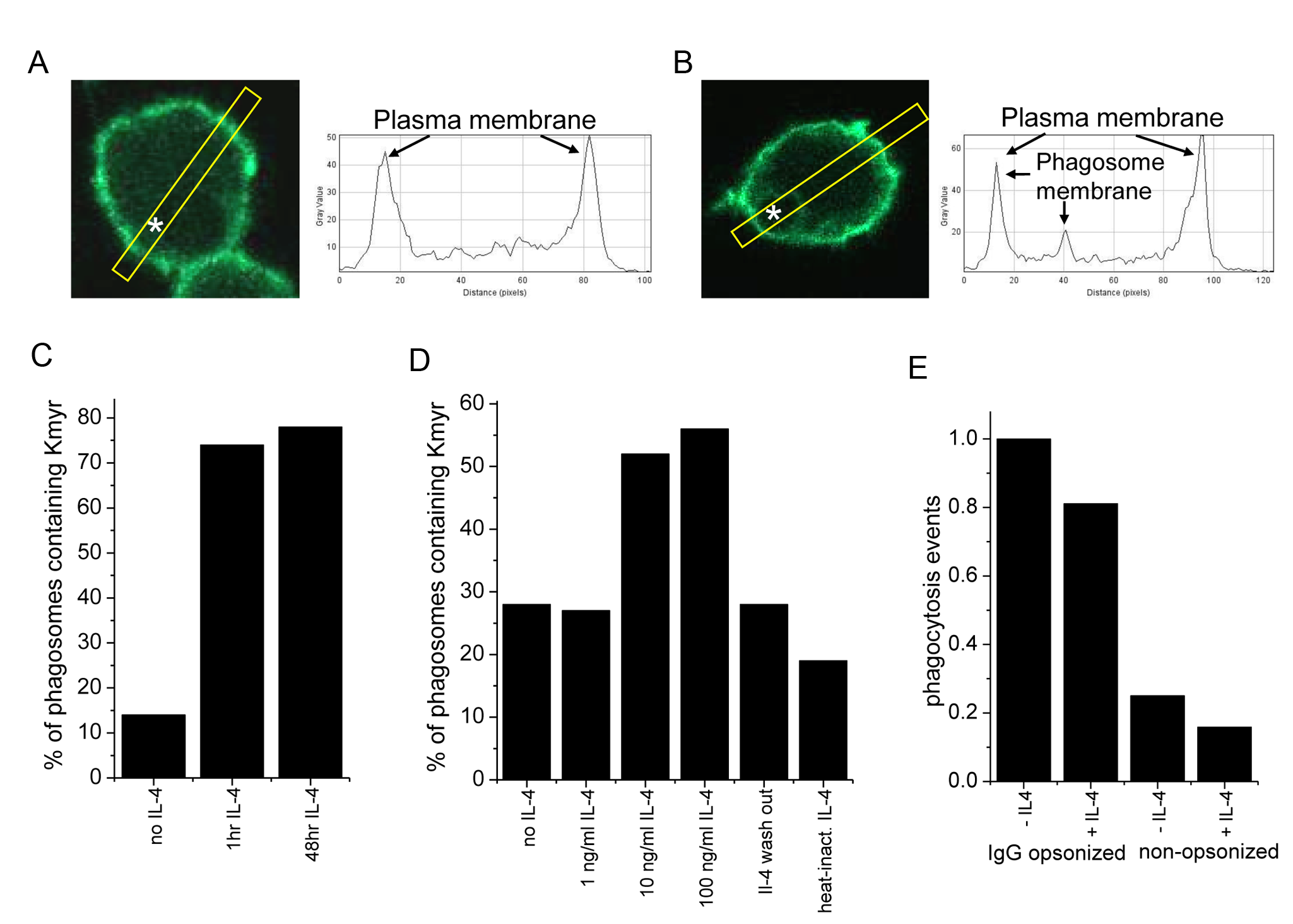

Supplement: Figure S3 — IL-4 effect on Kmyr distribution during phagocytosis. Serum starved MΦs stably expressing Kmyr-GFP were stimulated or not with IL-4 (10 ng/ml) and subsequently challenged with Alexa633-labelled IgG-opsonized zymosan (1∶10 ratio) at room temperature (at which temperature no phagocytosis occurs) for 30 min after which they were shifted to 37°C to synchronize phagocytosis. After 10 min at 37°C, the cells were quickly fixed in 4% PFA, mounted in anti-fading reagent. This time point was experimentally chosen to provide the optimal amount of early phagosomes in which we could compare the distribution of Kmyr in the absence and precence of IL-4. Kmyr-GFP distribution on the phagosome was analyzed by 3D confocal laser scanning microscopy to confirm internalization of the zymosan particle. The images are representative examples of untreated MΦs (A) or MΦs shortly exposed (1 hr) to IL-4 (10 ng/ml) (B) and were chosen from the Z-stack which had the optimal focus for the center cross-section of the phagosome. The position of the zymosan particle is indicated with *. The integrated fluorescence intensity values along the rectangle (10 pixels wide) crossing the cell in the image is plotted. Scale bar indicates 5 µm. (C) The number of Kmyr-GFP bearing phagosomes was determined as the fraction of total observed phagosomes ± SE in untreated or 1 hr IL-4 treated or 48 hrs IL-4 treated cells (* p<0.005 as determined by Fisher's exact test). (D) The number of Kmyr-GFP bearing phagosomes was determined as the fraction of total observed phagosomes ± SE in untreated cells or cells treated with either 1 ng/ml, 10 ng/ml and 100 ng/ml IL-4 (1 hr) (* p<0.005 as determined by Fisher's exact test). Kmyr-GFP bearing phagosomes were also monitored after washing away the IL-4 after 1 hr treatment (10 ng/ml) and allowing the cells recover for 1 hr, and upon treatment with heat-inactivated IL-4 (10 ng/ml, 1 hr). (E) The number of phagocytosis events of IgG-opsonized zymosan and non-opsonized zymosan [file pone.0022328.s003.tif]

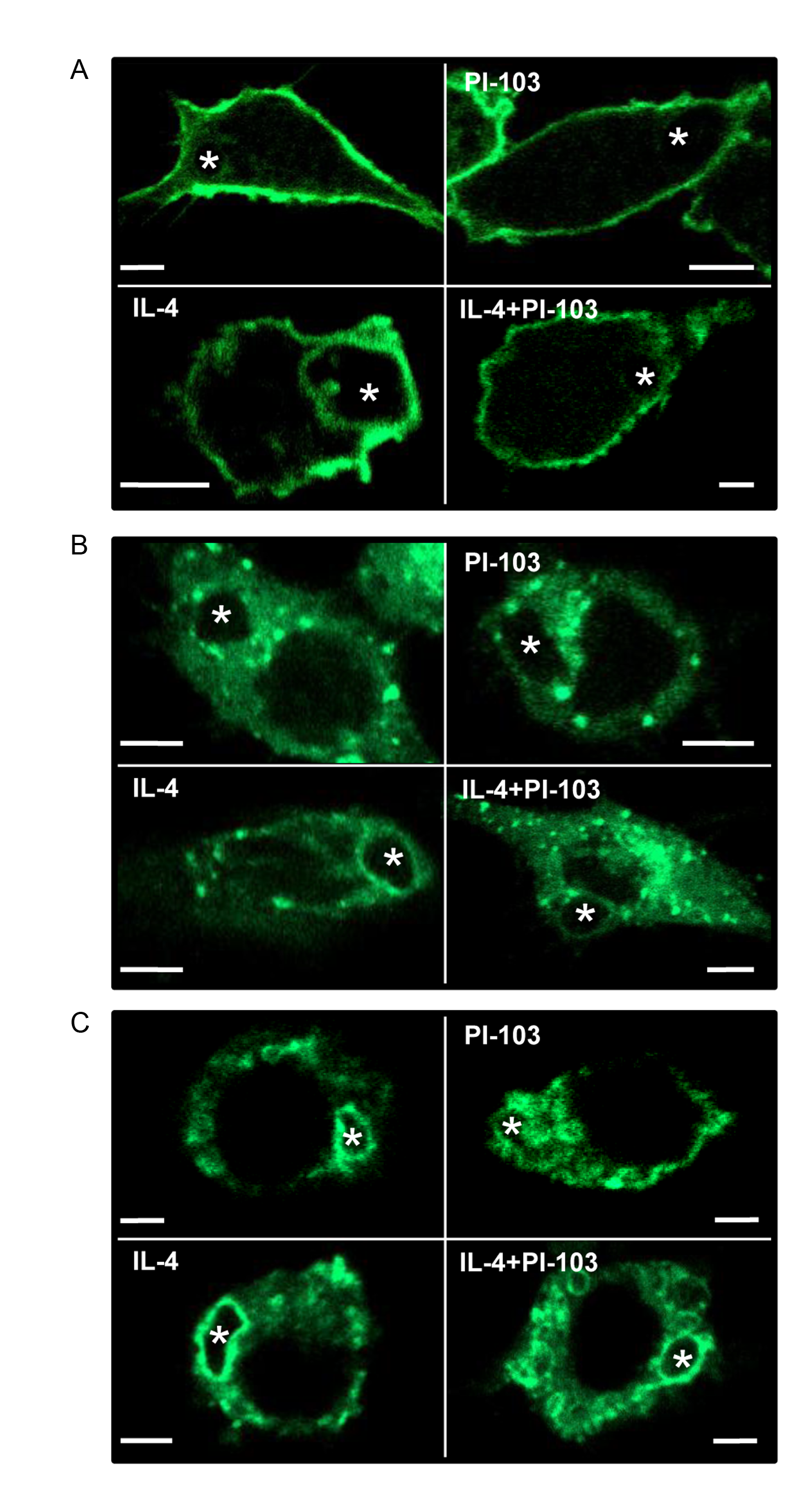

Supplement: Figure S4 — Blocking class I PI3K abrogates the IL-4 induced changes during phagocytosis. Serum starved MΦs stably expressing Kmyr-GFP (A), transiently expressing Rab5-GFP (B) or Rab7-GFP (C) were stimulated or not with IL-4 (10 ng/ml) and subsequently challenged with Alexa633-labelled IgG-opsonized zymosan (1∶10 ratio) at room temperature (at which temperature no phagocytosis occurs) for 30 min after which they were shifted to 37°C to synchronize phagocytosis. 5 min after the temperature shift PI-103 (100 nM), a specific class I PI3K inhibitor, was added. After 10 min at 37°C, the cells were quickly fixed in 4% PFA, mounted in anti-fading reagent, and Kmyr-GFP or Rab5-GFP distribution on the phagosome was analyzed by 3D confocal laser scanning microscopy. Scale bars indicate 3 µm. (TIF) [file pone.0022328.s004.tif]

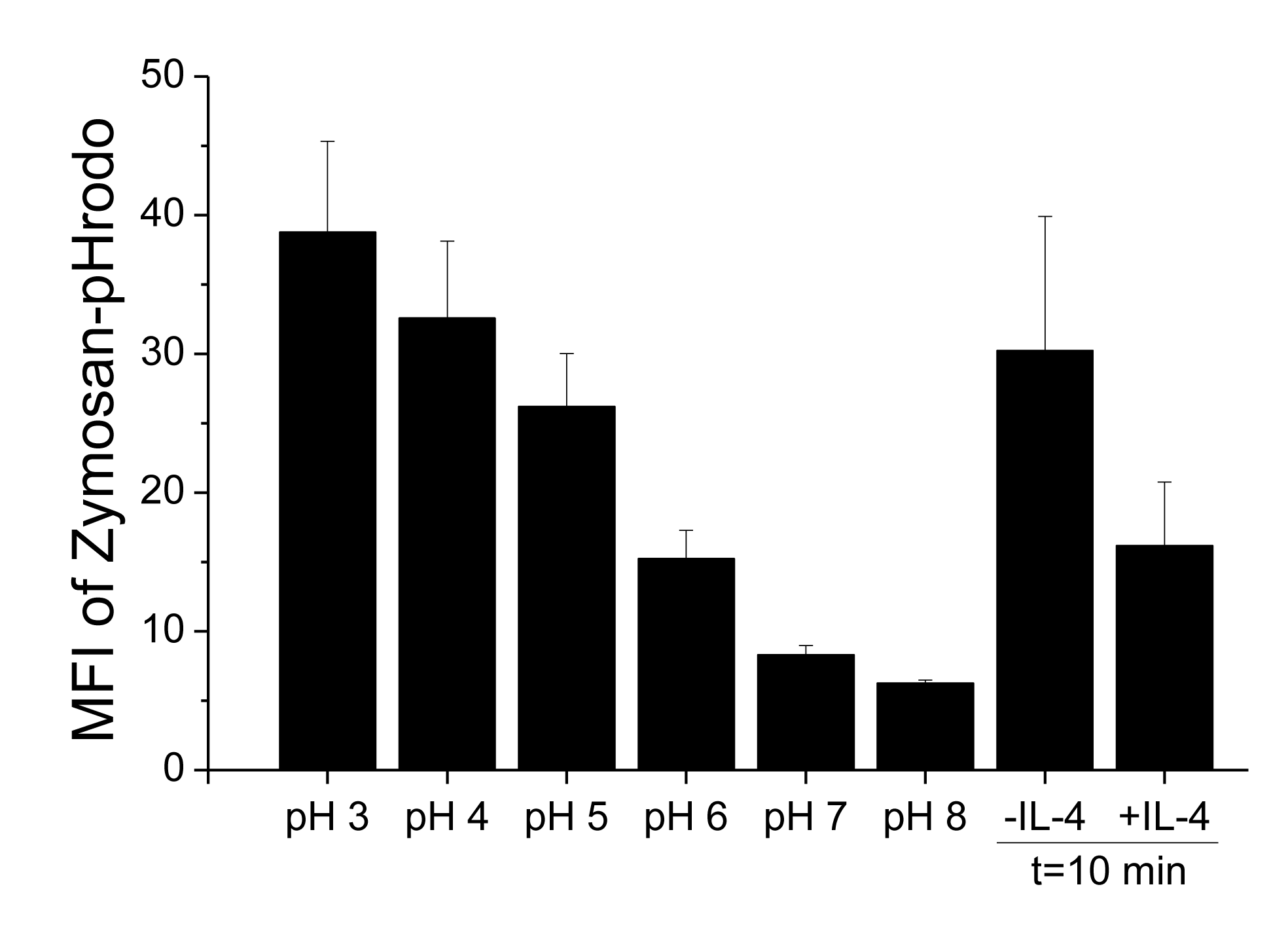

Supplement: Figure S5 — pH range of zymosan labeled with pHrodo. Zymosan particles labeled with the pH-sensitive dye pHrodo, which is nonfluorescent at neutral pH and fluoresces bright red in acidic environments, were placed on Poly-L Lysine coated Wilco dishes (Wilco dishes BV). Fluorescence of the same pHrodo-zymosan particles was monitored at different pH by 3D confocal microscopy and the mean fluorescence intensity of three cross-section of the pHrodo-zymosan particle from the Z-stack was calculated at each timepoint. The values at each pH represent the average +/− SD obtained from multiple pHrodo-zymosan particle (N = 30). The data were compared with the MFI obtained for pHrodo-zymosan containing phagosomes in MΦs untreated or shortly exposed (1 hr) to IL-4 (10 ng/ml) 10 min upon phagocytosis. (TIF) [file pone.0022328.s005.tif]
